# Comparative genomics of Toll-like receptor signalling in five species

**DOI:** 10.1186/1471-2164-10-216

**Published:** 2009-05-11

**Authors:** Oliver C Jann, Annemarie King, Nestor Lopez Corrales, Susan I Anderson, Kirsty Jensen, Tahar Ait-ali, Haizhou Tang, Chunhua Wu, Noelle E Cockett, Alan L Archibald, Elizabeth J Glass

**Affiliations:** 1 The Roslin Institute and R(D)SVS, University of Edinburgh, Roslin, Midlothian, Edinburgh, EH25 9PS, UK; 2 Public University of Navarra, Campus de Arrosadia s/n, 31006 Pamplona, Spain; 3 Department of Animal, Dairy, and Veterinary Sciences, Utah State University, Logan, UT 844322-4700 USA

## Abstract

**Background:**

Over the last decade, several studies have identified quantitative trait loci (QTL) affecting variation of immune related traits in mammals. Recent studies in humans and mice suggest that part of this variation may be caused by polymorphisms in genes involved in Toll-like receptor (TLR) signalling. In this project, we used a comparative approach to investigate the importance of TLR-related genes in comparison with other immunologically relevant genes for resistance traits in five species by associating their genomic location with previously published immune-related QTL regions.

**Results:**

We report the genomic localisation of *TLR1-10 *and ten associated signalling molecules in sheep and pig using *in-silico *and/or radiation hybrid (RH) mapping techniques and compare their positions with their annotated homologues in the human, cattle and mouse whole genome sequences. We also report medium-density RH maps for porcine chromosomes 8 and 13. A comparative analysis of the positions of previously published relevant QTLs allowed the identification of homologous regions that are associated with similar health traits in several species and which contain TLR related and other immunologically relevant genes. Additional evidence was gathered by examining relevant gene expression and association studies.

**Conclusion:**

This comparative genomic approach identified eight genes as potentially causative genes for variations of health related traits. These include susceptibility to clinical mastitis in dairy cattle, general disease resistance in sheep, cattle, humans and mice, and tolerance to protozoan infection in cattle and mice. Four TLR-related genes (*TLR1*, *6*, *MyD88*, *IRF3*) appear to be the most likely candidate genes underlying QTL regions which control the resistance to the same or similar pathogens in several species. Further studies are required to investigate the potential role of polymorphisms within these genes.

## Background

The innate immune system is the first line of defence against invading pathogens and is activated by conserved pathogen associated molecular patterns (PAMPs). Toll-like receptors (TLRs), a family of signalling molecules that bind to PAMPs and consequently trigger an immune response [1], play a major role within the innate immune system. TLRs are found in all animals and even plant homologues have been described [2], illustrating the ancient origin of this gene family. Most mammalian species share ten TLR genes (*TLR1-10*), each detecting PAMPs with different molecular structures.

TLRs bind their ligands in a horseshoe-shaped leucine rich repeat (LRR) domain, which enables a Toll/interleukin-1 receptor (TIR) domain to associate with adapter proteins like the Toll/interleukin-1 receptor domain-containing adapter protein (TIRAP), lymphocyte antigen 96 (LY96 or MD2), or myeloid differentiation primary response protein (MyD88) which binds with the interleukin-1 receptor-associated kinase 1 (IRAK-1). This binding activates the tumour necrosis factor receptor-associated factor 6 (TRAF6), triggering a cascade which finally results in nuclear factor-kappa B (NF-κB) liberation, activating the expression of pro-inflammatory genes (reviewed by Werling & Jungi [3]). An additional molecule, the Toll-interacting protein (TOLLIP), is involved in the regulation of this process [4]. MyD88, TIRAP, IRAK-1 and TRAF6 are also involved in TLR-induced apoptosis mediated by caspase-8 (CASP8) (reviewed by Bannerman & Goldblum [5]). The toll-like receptor adaptor molecules (TICAM-1 or TRIF and TICAM-2 or TRAM) have been shown to activate TRAF6 and also to trigger interferon α or β (IFN-α/β) responses [6,7]. The transcriptional regulation of type I interferons is coordinated, at least in part, by interferon regulatory factors 3 and 7 (IRF3/7). IRF3 and IRF7 can also be activated by kinases which are regulated by MyD88/TRAF6 [8]. The pre-eminence of TLRs and these associated signalling molecules in the initial recognition of pathogens suggests that they could be strong candidates for animal health traits.

In humans, polymorphisms within genes coding for TLR and associated signalling molecules are associated with a predisposition to several diseases [9-11]. There is increasing evidence pointing to the strong possibility that polymorphisms in livestock TLR genes might affect immune related traits [12-14] and might explain at least part of the observed variation in disease resistance. A number of immune-related quantitative trait loci (QTL) studies have been conducted in the major livestock species and the data are made publicly available [15,16]. However, the causal genes underlying these QTLs have not been identified. Consequently, the TLR genes and their related signalling molecules which are located within these QTLs should be considered as potential candidates for explaining phenotypic variation in disease related traits and could therefore be exploited through genetic selection for desirable alleles.

Another approach to identify genes underlying variation in immune responses is the analysis of gene expression patterns in populations with divergent resistance status *pre *or *post *infection. In mice, several differential gene expression studies involving a multitude of traits have been conducted, resulting in large datasets which are publicly available [17,18]. However, this type of information is more limited for livestock species. Differential expression of *TLR*s and related genes has been analysed in the gastrointestinal tract of sheep infected with *Haemonchus contortus *and *Trichostronglyus colubriformis *[19]. In cattle, expression differences have been investigated in breeds of different susceptibility to *Theileria annulata *[20] and *Trypanosoma congolense *(Kemp, personal communication). Studies in pig have mostly addressed the role of specific TLRs during host pathogen interaction and have been reviewed recently [21]. However, to date, no studies in pigs have been undertaken to investigate TLR gene expression differences in phenotypically divergent lines.

Although genes involved in TLR signalling have been annotated in the mouse and human genomes and successfully mapped in cattle [22,23], only a subset of *TLR*s and no TLR-associated signalling molecules have been localised in other livestock species. Only *TLR2*, *TLR4*, *TLR6 *and *TLR9 *have been mapped in the porcine genome [24,25], while the locations of the sheep *TLR *and associated genes are currently unknown.

Here we report the genomic locations of ten TLR genes (*TLR1-10*) and a further ten associated signalling molecules in sheep, pig, cattle, human and mouse and compare their positions with previously published health related QTLs. We identify TLR-related genes which are located in homologous regions that are associated with similar health related traits in several species and investigate their importance by functional comparison with other linked immune related genes.

## Results

### Localisation of TLR and signalling genes in the pig genome

The Roslin-Cambridge porcine RH panel [26] was screened with six TLR-related genes, 20 other genes and 72 microsatellite markers, all predicted to be on porcine chromosomes 8 and 13, based on comparative analysis of the pig fingerprinted contig (FPC) map [27].

#### RH map of pig chromosome 8

Fifty-nine markers (23 genes and 36 microsatellites) were assigned to five linkage groups at LOD4 on porcine chromosome 8 (SSC8), which themselves were ordered into two groups corresponding to both arms (SSC8a with 24 markers and SSC8b with 35 markers) of the chromosome using markers in common with the MARC v2 porcine linkage map [28] as the scaffold (Additional file 1: Pig chromosome 8). The length of SSC8a and b was 915.2 centiray (cR) and 1312.9 cR, respectively. The resulting RH maps showed a very consistent marker order when compared to the MARC v2 map (Additional file 1: Pig chromosome 8). The here created maps are publicly available in the Arkdb database [29]. Four TLR genes were assigned to this chromosome; *TLR1*, *6 *and *10 *are closely linked between 446.9 and 490.5 cR on SSC8a, whereas *TLR2 *maps at 310.5 cR on SSC8b (Additional file 1: Pig chromosome 8).

#### RH map of pig chromosome 13

Thirty-nine markers (three genes and 36 microsatellites) were assigned to 14 linkage groups at LOD4 on porcine chromosome 13 (SSC13), which themselves were ordered along the chromosome using markers in common with the MARC v2 map [28] as the scaffold. The total map length was 2669.0 cR.

Comparison with the MARC v2 map displays a very consistent marker order (Additional file 2: Pig chromosome 13). The resulting RH map is now publicly available at the ArkDB database [29]. *MyD88 *was located on this chromosome at 354.4 cR and *TLR9 *at 588.8 cR.

#### Comparison with the porcine FPC map

Eighteen of the 20 *TLR *and associated signalling genes could be localised using comparative information between the porcine FPC map and the human whole genome sequence [30]. Two genes (*TICAM1 *and *TOLLIP*) could not be assigned to a position in the porcine FPC map because a 3 Mb human sequence fragment surrounding the localisation of the genes produced no significant alignment with any porcine clone mapped on the FPC map. Of the 18 genes with predicted locations, six (*TLR1, 2, 6, 10 *on SSC8 and *TLR9 *and *MyD88 *on SSC13) were mapped on the porcine RH map using the Cambridge-Roslin RH panel (Table 1). The positions for *TLR4 *on SSC1 and *TLR9 *on SSC13 on the FPC map agree with another study [25]. Thus, *in-silico *positions were confirmed by lab based mapping techniques for seven of the 18 TLR-related genes (Table 1). In addition, an alignment of publicly available porcine mRNA sequences of the genes against the current pre-assembled HTGS (high throughput genomic sequence) pig sequence database [31] resulted in 14 alignments which all confirmed the positions predicted by the FPC map (Table 1).

**Table 1:** Location of TLR and related signalling genes on the porcine FPC map compared to the porcine RH map

	**FPC map**		**HTGS**	**RH map**			**Further map information**
**Gene**	**SSC**	**Position**	**SSC**	**SSC**	**LOD**	**Marker**	

*TLR1*	8	30.3	8	8	9.0	*TLR6*	
*TLR2*	8	83.6		8	5.8	*S086*	
*TLR3*	15	58.9	15				
*TLR4*	1	284.7	1				SSC1q2.9-q2.13 [25]
*TLR5*	10	14.9	10				
*TLR6*	8	30.3	8	8	13.9	*TLR10*	
*TLR7*	X	9.3					
*TLR8*	X	9.4					
*TLR9*	13	39.7	13	13	4.2	*SW864*	SSC13q2.1-q3.2 [25]
*TLR10*	8	30.3	8	8	14.1	*SW444*	
*CASP8*	15	128.6	15				
*IRAK-1*	X	133.9	X				
*LY96*	4	70.7	4				
*MyD88*	13	29.0	13	13	9.2	*S0288*	
*TICAM1*							
*TICAM2*	2	120.8					
*TIRAP*	9	57.0	9				
*TOLLIP*							
*TRAF6*	2	22.8	2				
*IRF3*	6	52.8	6				

HTGS: BLAST hits against the high throughput genomic sequence of the pig

### Localisation of TLR and signalling genes in the sheep genome

#### In-silico and radiation hybrid mapping in sheep

All 20 TLR-related genes could be localised in the virtual sheep genome [32,33]. In order to confirm these *in-silico *positions (Table 2), primers for the 20 genes (Additional file 3: Primers used for RH mapping of TLR and signalling molecules) were used to screen the USU oRH5000 ovine radiation hybrid panel [34] to analyse linkage with previously assigned markers on the ovine RH map. Significant linkage was demonstrated for 16 of the 20 loci by LOD scores greater than 5, allowing an assignment of the loci to the ovine RH map and a comparison to the *in-silico *position predicted by the virtual sheep genome (Table 2).

**Table 2:** Location of TLR and related signalling genes on the virtual sheep genome compared to the ovine RH map

	**Virtual genome**		**RH map**		
**Gene**	**OAR**	**Position**	**OAR**	**LOD**	**Marker**

*TLR1*	6	55.5	6	5.60	*MCMA9*
*TLR2*	17	3.7	17	15.36	*MNS101B*
*TLR3*	26	18.6	26	6.69	*RM209*
*TLR4*	2	3.7	2	11.57	*CSSM47*
*TLR5*	12	38.7	12	8.62	*TGLA53*
*TLR6*	6	55.5	6	10.25	*BMS483*
*TLR7*	X	12.7	X	9.36	*TLR8*
*TLR8*	X	12.7	X	9.36	*TLR7*
*TLR9*	19	52.9	19	14.16	*BMS693*
*TLR10*	6	55.5	6	13.55	*KLHL1*
*CASP8*	2	228.8	[1]	3.70	*UROD*
*IRAK-1*	X	96.1	[unlinked]	2.47	*GDI1*
*LY96*	9	67.8	9	7.26	*CL634047*
*MyD88*	19	10.8	19	11.07	*BM1558*
*TICAM1*	5	23.1	5	7.50	*MAP2K2*
*TICAM2*	5	43.0	[7]	2.97	*BMS2614*
*TIRAP*	21	25.6	21	7.49	*JP15*
*TOLLIP*	21	47.9	[21]	2.49	*BMS1948*
*TRAF6*	15	62.1	15	5.38	*ILSTS27*
*IRF3*	14	78.4	14	9.31	*LHBP16*

Markers marked by [ ] have LOD scores < 5

The remaining four genes were linked to markers on the ovine RH map but with LOD scores of ≤ 5.0. One of these four genes (*TOLLIP*) was tentatively linked to a marker on chromosome 21 (LOD = 2.49), the same location predicted by the virtual sheep genome. Therefore, while the LOD score for the RH mapping was not significant, the RH analysis supported the *in-silico *position (Table 2). Three genes were tentatively assigned on the RH map to locations other than predicted by the virtual sheep genome (*TICAM2 and CASP8*) or unlinked to any other marker (*IRAK-1*) but all three had non-significant LOD scores (2.97, 3.7, and 2.97, respectively), suggesting that the location predicted by the virtual sheep genome was more plausible than the RH location (Table 2).

In summary, the predicted positions of 17 genes on the virtual sheep genome were confirmed (LOD ≥ 5.0) or supported (LOD = 2.49) by RH mapping. The remaining three genes were not positioned with confidence on the RH map so the positions predicted by the virtual sheep genome could not be confirmed.

### Homologous regions affecting related traits in several species

Genomic coordinates of the TLR-related genes were compared with the locations of health-related QTLs in pig, sheep, cattle, human and mouse (Table 3, [35-71]). Six of the analysed genes are located in homologous QTL regions which control the susceptibility to the same or a closely related pathogen in several species. Five of them (*TLR1, 6, 9, MyD88 *and *IRF3*) are functionally involved in immune responses against the QTL associated pathogens (Table 3). In addition association studies have linked polymorphic variants of human and murine TLR1, 6 and IRF3 with susceptibility to relevant diseases. Further evidence arises also for MyD88 by differential expression in mouse strains of divergent resistance post infection with *Trypanosoma congolense *which is of particular interest because of the ambiguous involvement of MyD88 into the control of protozoan infections [72].

**Table 3:** Comparative localisation of TLR and related signalling molecules

	**Pig**		**Sheep**		**Cattle**		**Mouse**		**Human**	
**Gene**	**Position**	**QTL**	**Position**	**QTL**	**Position**	**QTL**	**Position**	**QTL**	**Position**	**Associations**

***TLR1***	8: 30.3		6: 55.5		6: 60.4	7	5: 65.3	12, 15, 16	4: 38.5	m, g'
***TLR2***	8: 83.6	1	17: 3.7		17: 4.3		3: 83.6	12, 13, 17	4: 154.8	a, b, c, d, e
***TLR3***	15: 58.9		26: 18.6		27: 17.5		8: 46.5	12	4: 187.2	
***TLR4***	1: 284.7		2: 3.7		8: 112.4		4: 66.5	18	9: 119.5	f, g, h
***TLR5***	10: 24.9		12: 38.7		16: 23.6	10	1: 184.9	12, 16, 17, 19	1: 221.3	i
***TLR6***	8: 30.3		6: 55.5		6: 60.4	7	5: 65.3	12, 15, 16	4: 38.5	m, g'
***TLR7***	X: 9.3		X: 12.7		X: 82.1		X: 163.7		X: 12.8	
***TLR8***	X: 9.4		X: 12.7		X: 82.0		X: 163.7		X: 12.8	
***TLR9***	13: 39.7		19: 52.9		22: 49.7	9	9: 106.1	18	3: 52.2	g'
***TLR10***	8: 30.3		6: 55.5		6: 60.3	7	n/a: n/a		4: 38.4	
***CASP8***	15: 128.6				2: 94.0		1: 58.8	18, 20	2: 201.8	
***IRAK-1***	X: 133.9				X: 23.5		X: 71.3		X: 152.9	f'
***LY96***	4: 70.7		9: 67.8		14: 35.0	9'	1: 16.7	17	8: 75.1	
***MyD88***	13: 29.0		19: 10.8		22: 11.7	10	9: 119.2	18, 21	3: 38.2	
***TICAM1***			5: 23.1		7: 17.9		17: 56.4	22	19: 4.7	11
***TICAM2***	2: 120.8	2			10: 3.9		18: 46.7	12, 23	5: 114.9	
***TIRAP***	9: 57.0		21: 25.6		29: 31.2		9: 35.0	14, 23	11: 125.7	g', j, k, m'
***TOLLIP***			21: 47.9		29: 44.0		7: 149.1	24	11: 1.3	
***TRAF6***	2: 22.8		15: 62.1		15: 62.1	8	2: 101.5		11: 36.5	
***IRF3***	6: 52.8	3,4,5, l	14: 78.4	6	18: 56.0	7'	7: 52.3	13, n	19: 54.8	11

n/a: no functional homologue for *TLR10 *in mouse,

**QTL studies**: 1: Stress induced alteration in number of neutrophils [35], 2: Stress induced leukocyte proliferation [35], 3: Small intestinal *Escherichia coli *[36], 4,5: Anti 0149 *Escherichia coli *IgG levels/level response [35], 6: *Nematodirus *FEC1 Average [37], 7: Clinical mastitis [38], 7': Clinical mastitis [39], 8: General disease resistance [40], 9: Somatic cell score [41], 9': Somatic cell score [42], 10: *Trypanosoma congolense *tolerance [43], 11: Coxsackie virus B3 sensitivity [44], 12: *Leishmania *resistance [45], 13: *Mycobacterium tuberculosis *susceptibility [46], 14: *Mycobacterium tuberculosis *infection severity [47], 15: *Listeria monocytogenes *resistance [48], 16: *Trypanosoma cruzi *infection response [49], 17: Theiler's murine encephalomyelitis virus induced demyelinating disease susceptibility [50], 18: *Borrelia burgdorferi*-associated arthritis [51], 19: *Plasmodium berghei *malaria resistance [52], 20: Susceptibility/immunity to *Salmonella typhimurium *antigens [53], 21: *Plasmodium chabaudi *malaria resistance [54], 22: *Angiostrongylus costaricensis *nematode susceptibility [55], 23: Protection against vaginal *Candida albicans *infections [56], 24: Determination of interleukin commitment [57]

**Association studies**: a: *Mycobacterium sp. *[58], b: *Mycobacterium leprae *[59], c: Urinary tract infections [60], d: *Borrelia burgdorferi *[61], e: *Treponema pallidum *[62], f: Gram-negative infections [63], f': Sepsis [64], g: *Plasmodium falciparum *[65], g': *Plasmodium falciparum *[66], h: Bacterial vaginosis [67], i: *Legionella pneumophila *[68], j: Bacteremia [69], k: *Pneumococcia sp. *[69], l: Small intestinal *Escherichia coli *[36], m: *Mycobacterium tuberculosis *[70], m': *Mycobacterium tuberculosis *[69], n: *Listeria monocytogenes *[71]

**Figure 1:**
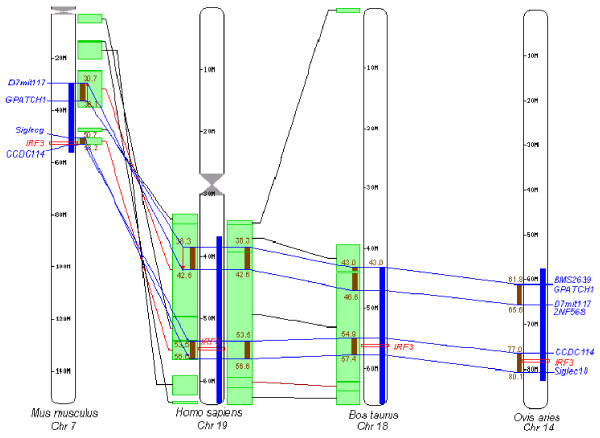
**Position of *IRF3 *and overlap of QTLs in mouse, human, cattle and sheep**. QTL positions are indicated by bold blue lines. Green boxes indicate the localisation of syntenic blocks conserved between species. Inversions of the gene order are indicated by red arrows. Markers located on the boundaries of the QTLs in mouse (susceptibility to *Mycobaterium tuberculosis*), human (Coxsackie virus resistance), cattle (susceptibility to clinical mastitis) and sheep (*Nematodirus *egg count) or the blocks of conserved synteny are indicated in blue. Under the assumption that the indicated QTLs are caused by the same loci, the significant region can be narrowed to two segments with a combined length of less than 7 Mb (brown line in syntenic blocks). Immunologically relevant genes located in these regions are listed in additional file 4: Immunologically relevant genes in regions of conserved synteny surrounding the *TLR1 *family cluster, *MyD88 *and *IRF3*.

**Figure 2:**
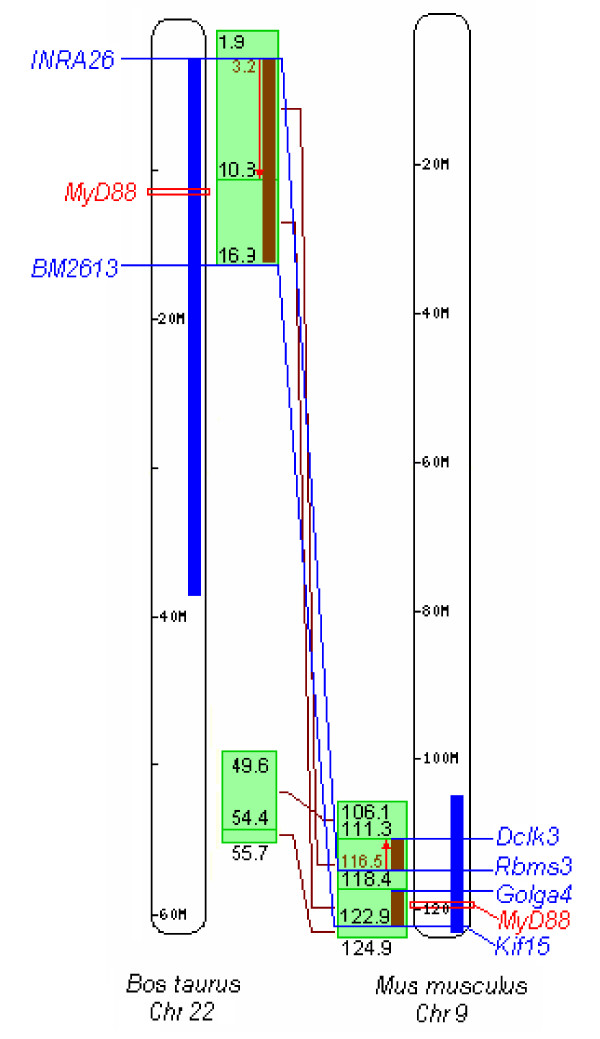
**Position of *MyD88 *and overlap of QTLs in cattle and mouse**. QTL positions are indicated by bold blue lines. Green boxes indicate the localisation of syntenic blocks conserved among species. Inversions of the gene order are indicated by red arrows. Loci located on the boundaries of the overlap between the QTL in cattle (*Tryanosoma *resistance) and in mice (*Plasmodium chabaudi *malaria) are indicated in blue. Under the assumption that the indicated QTLs are caused by the same loci, the significant region can be narrowed to segments with a combined length of approximately 10 Mb (brown line in syntenic blocks). Immunologically relevant genes located in these regions are listed in additional file 4: Immunologically relevant genes in regions of conserved synteny surrounding the *TLR1 *family cluster, *MyD88 *and *IRF3*.

**Figure 3:**
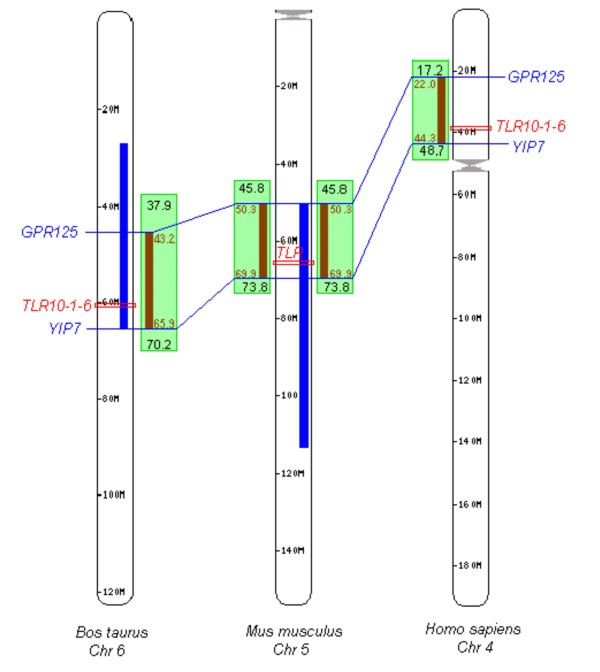
**Position of the *TLR1 *family cluster and overlap of QTLs in cattle, mouse and human**. QTL positions are indicated by bold blue lines. Green boxes indicate the localisation of the syntenic block conserved between species. Under the assumption that the indicated QTLs are caused by the same loci, the significant region can be narrowed to a segments with a length of approximately 20 Mb (brown line in syntenic blocks). Two loci (*GPR125 *and *YIP7*, in blue) limit the overlap of the QTL for susceptibility to clinical mastitis in cattle with the QTL for *Listeria monocytogenes *susceptibility in mice. Polymorphisms in human *TLR6 *(red, within the QTL overlap) have been associated with susceptibility to tuberculosis. Immunologically relevant genes located in this region are listed in additional file 4: Immunologically relevant genes in regions of conserved synteny surrounding the *TLR1 *family cluster, *MyD88 *and *IRF3*.

These four genes are located in QTL regions which harbour further immunologically relevant genes. Assuming that homologous QTLs are controlled by the same genes in several species, the QTL span was narrowed down to the common block of conserved gene synteny among the species (Figures 1, 2 and 3).

*IRF3 *is located in a region affecting health traits in all five species, but the QTL controls host responses for a wide range of pathogens (Table 3). The homologous QTL overlap among mouse, human, cattle and sheep comprises two blocks of conserved gene synteny between these species and has a combined extent of approximately 7 Mb (Figure 1). The region contains in human, mouse and cattle 241, 263 and 210 genes, respectively. Seventy-seven of them are listed in the innatedb non-redundant gene list of immune-related murine or humane genes [73] (Additional file 4: Immunologically relevant genes in regions of conserved synteny surrounding the *TLR1 *family cluster, *MyD88 *and *IRF3*). Eight genes were considered as functionally relevant according to their gene ontology (GO) annotation (Table 4).

**Table 4:** Potential QTL related candidate genes with functional relevance, differential expression in divergent phenotypes, and localization within QTL regions

**Gene**	**Human**	**Mouse**	**Cattle**	**Relevant function**	**Mouse**	**Mouse**	**Sheep**	**Cattle**
	**Chr: Mb**	**Chr: Mb**	**Chr: Mb**		**Y.e.**	**T.c.**	**H.c.**	**T.a.**
***GPI***	19: 39.5	7: 35.0	18: 44.5	humoral immune response		n.a.	n.a.	n.a.
***HAMP***	19: 40.5	7: 31.7	18: 45.4	antimicrobial activity of HAMP derived peptides			n.a.	n.a.
***CD22***	19: 40.5	7: 31.7	18: 45.5	inhibition of B cell receptor signalling		2.24*e	n.a.	n.a.
***TYROBP***	19: 41.1	7: 31.2	18: 46.0	activation of NK cells		2.11**e	n.a.	
***FUT1***	19: 53.9	7: 52.9	18: 55.2	creation of an adhesion site			n.a.	n.a.
***FCGRT***	19: 54.7	7: 52.3	18: 55.9	IgG fragment receptor	0.73**b		n.a.	n.a.
***IRF3***	19: 54.9	7: 52.3	18: 56.0	activation of IFN-β		1.22*d		n.a.
***PRMT1***	19: 54.9	7: 52.2	18: 56.0	inhibition of viral helicase			n.a.	n.a.

***CCR4***	3: 33.0	9: 114.4	22: 7.3	chemokine receptor	i.s.		n.a.	n.a.
***MYD88***	3: 38.2	9: 119.2	22: 11.7	mediation of signal after TLR-ligand binding		1.39*d		
***CX3CR1***	3: 39.3	9: 120.0	22: 12.8	chemokine receptor	0.54**a	2.09*c	n.a.	
***CCR8***	3: 39.3	9: 120.0	22: 12.8	chemokine receptor	i.s.		n.a.	n.a.
***VIPR1***	3: 42.5	9: 121.6	22: 15.0	binding of anti-inflammatory peptide	i.s		n.a.	n.a.
***CCBP2***	3: 42.8	9: 121.8	22: 15.3	chemokine receptor	i.s.	1.53*e	n.a.	n.a.

***TLR10***	4: 38.5	n.o.	6: 60.3	binding of unknown ligand	n.o.	n.o.	2.40*	1.71*
***TLR1***	4: 38.5	5: 65.3	6: 60.4	binding of ligands derived from gram-positive bacteria		1.37*c		
***TLR6***	4: 38.5	5: 65.3	6: 60.4	binding of ligands derived from gram-positive bacteria		0.61*d		
***RFC1***	4: 39.0	5: 65.7	6: 60.8	replication factor C (activator 1) 1,	defence response		0.50*b	n.a.

human/mouse/cattle: Positions in Mb, only significant values shown, empty cells have been analysed and no significant difference have been found. *: p < 0.05, **: p < 0.01, i.s: signal was evaluated as too faint to call, n.o: no mouse ortholog for TLR10, n.a: not analysed in this study

**Gene names**: *GPI*: glucose phosphate isomerase, *HAMP*: hepcidin antimicrobial peptide, *CD22*: CD22 molecule, *TYROBP*: *TYRO *protein tyrosine kinase binding protein, *FUT1*: fucosyltransferase 1, *FCGRT*: IgG Fc fragment receptor transporter alpha chain, *IRF3*: interferon regulatory factor 3, *PRMT1*: protein arginine methyltransferase 1, *CCR4*: chemokine (C-C motif) receptor 4, *MYD88*: myeloid differentiation primary response gene (88), *CX3CR1*: chemokine (C-X3-C motif) receptor 1, *CCR8*: chemokine (C-C motif) receptor 8, *VIPR1*: vasoactive intestinal peptide receptor 1, *CCBP2*: chemokine binding protein 2, *TLR10*: toll-like receptor 10, *TLR1*: toll-like receptor 1, *TLR6*: toll-like receptor 6, *RFC1*: replication factor C (activator 1) 1

**Ratios of transcript levels**: a: ratio of mean transcript levels of resistant C57BL/6 to susceptible BALB/c mice 3 hours post infection and stimulation with IFN-γ, b: ratio of mean transcript levels of resistant C57BL/6 to susceptible BALB/c mice 3 hours post infection without stimulation with IFN-γ, c: ratio of mean transcript levels of resistant C57BL/6 to susceptible BALB/c mice on day 3 *post *infection, d: ratio of mean transcript levels of resistant C57BL/6 to susceptible A/J mice on day 9 *post *infection, e: ratio of mean transcript levels of resistant C57BL/6 to susceptible A/J mice in uninfected animals

**Pathogens and references**: Y.e: *Yersinia enterocolitica *[86], T.c: *Trypanosoma congolense *[85], H.c: *Haemonchus contortus *[19], T.a: *Theileria annulata*: transcript level in Holstein/Sahiwal 72 h post infection [20]

*MyD88 *is located in a QTL related to protozoan infections in cattle and mice (Table 3). There are four regions of conserved synteny between bovine chromosome 22 and murine chromosome 9 which are differentially ordered and orientated between both species. Together they comprise approximately 10 Mb, within which the parasite-related QTLs in mouse and cattle overlap (Figure 2). Both QTLs share an area which in cattle and mouse comprises 97 and 100 genes, respectively. Thirty-eight genes are listed in the innatedb non-redundant gene list of immunologically relevant murine or human genes [73] (Additional file 4: Immunologically relevant genes in regions of conserved synteny surrounding the *TLR1 *family cluster, *MyD88 *and *IRF3*) and six were considered as functionally relevant according to their GO annotation (Table 4).

The homologous QTL regions overlaying the *TLR1 *family cluster controls bacterial infections in three species (Table 3). Their 20 Mb overlap region (Figure 3) contains in human, cattle and mouse 68, 63 and 71 genes, respectively. Out of those 16 genes are listed in the innatedb non-redundant gene list of immunologically relevant murine or bovine genes [73] (Additional file 4: Immunologically relevant genes in regions of conserved synteny surrounding the *TLR1 *family cluster, *MyD88 *and *IRF3*).

The GO annotation indicates that four of them are involved in immune responses and therefore might be functionally relevant (Table 4).

## Discussion

### Reliability of the pig FPC map

It was the aim of this study to use information from different sources to infer the location of 20 porcine TLR-related genes. The gene content of the pig bacterial artificial chromosome (BAC) clones predicted on the basis of BES (BAC end sequence) alignments with the human genome has been validated by subsequent sequencing of BACs in the pig genome project [31], suggesting that the FPC map is a solid tool to identify gene locations. In addition to the *in-silico *information deduced from the pig FPC map [27] and the BLAST analysis of the pig HTGS sequence database [31] we also determined the location of several of the genes using the Roslin-Cambridge porcine radiation hybrid panel [26]. We performed the RH analysis on porcine chromosomes 8 and 13 because these two chromosomes were expected to harbour six genes of which the position of five is of particular interest. The common location of *TLR2 *with the *TLR1 *family cluster on one chromosome is unique to pig and human. The molecules of the TLR1 family (TLR1, 6, 10) broaden their ligand spectrum by heterodimerisation with TLR2 which is then signalled via a MyD88 dependent pathway [74]. It is striking that in human and pig these closely interacting molecules are linked together, whereas in other species they are on different chromosomes. This merits a more detailed analysis of the involved genomic region.

The high consistency of the marker order between the MARC v2 [28] and RH maps (additional files 1 and 2) and the confirmation of the predicted positions with all six genes mapped using RH techniques confirms that the FPC map is a reliable source of mapping information.

### Reliability of the virtual sheep genome

The virtual sheep genome [33] has been established by aligning BAC end sequence data from the CHORI-243 ovine BAC library against the sequences from the human (build hg17), bovine (build 2.0) and canine (build canFam2) genomes, and anchoring those with the ovine linkage map (version 4.6). The three inconsistencies between the virtual sheep genome and RH positions in this study (*CASP8*, *IRAK-1*, *TICAM2*) are likely due to limited loci on the RH map resulting in non-significant linkage, which could be resolved in the near future by adding additional markers to the RH map. Thus, the confirmation of 17 of the 20 genes by RH mapping using the USUoRH5000 panel [34] suggests that the *in-silico *approach for predicting gene positions using the virtual sheep genome holds great promise.

### Combined approach to identify health related candidate genes

Particularly in mice a multitude of health related QTLs cover relatively large proportions of the chromosomes so that the localisation of the genes within such a region provides rather limited evidence for an involvement into the mechanisms shaping the variation of the trait. However, homologous QTL regions can be narrowed by including comparative information from other species [75,76]. The more the size of the inter-specific QTL region can be reduced, the greater becomes the support for the candidate genes within this region.

The relationships of a gene to a phenotype can also be indicated by its functional relevance or by expression patterns which differ among phenotypes. Hence we pursue a combined approach which can provide much stronger evidence.

Genes within homologous QTL overlaps can be selected based on their ontology. Functional relevance has often been deduced from other species. This approach is not always reliable, as gene functions might change during evolution leading to limited differences among species [77]. For example in mice 12 TLRs are known [78], but only 9 of the TLR1-10 which are common in most mammals are functional in mice. However, the functional differences between mammals are relatively small and it can therefore be assumed that the gene function established in one mammalian species can in most cases be extrapolated to the others.

In addition differential expression patterns indicate that the genes might be involved in the mechanism(s) resulting in phenotypic differences, either as a consequence of a polymorphism in an upstream gene or in the gene itself. However, genes can also be associated with a divergent phenotype without a related difference in expression. Also, differential expression patterns do not necessarily indicate a direct involvement of a gene in the phenotype.

Therefore a combination of approaches is necessary and can provide much stronger evidence for or against the involvement of candidate genes in variations of disease resistance traits (Table 4).

### QTL regions and candidate genes

#### *IRF3 *and linked genes

The wide range of pathogens controlled by the QTL seems to suggest that different genes might be responsible for the QTL effect in the different species. In the pig, it is likely that the QTL affecting *Escherichia *(*E.) coli *resistance is caused by a polymorphism in the *FUT1 *(fucosyltransferase 1) gene which is closely linked to *IRF3 *[36]. The FUT1 enzyme modifies a structure that enables specific binding of *E. coli *(ECF18) to the intestinal mucosa and therefore could not explain the QTL effects on the non-bacterial pathogens in the other species (Table 3).

For *TYROBP *(TYRO protein tyrosine kinase binding protein) some evidence for adaptive selection within cattle populations has been found [79], indicating that polymorphism might influence resistance traits in cattle. TYROBP activates natural killer (NK) cells and therefore plays an important role in anti-viral defence [80] and could explain the human Coxsackie virus resistance locus, but not the overlapping QTLs in the other species.

To date only polymorphisms in *IRF3 *and *FCGRT *(IgG Fc fragment receptor transporter alpha chain) have been associated with relevant health traits. The FCGRT binds IgG, and serves to transfer IgG to mucosal surfaces. In ruminants and pigs it is likely to be particularly important in colostral immunoglobulin transfer to newborns as it is expressed in the newly lactating mammary gland. However, it is also expressed in adult mammalian tissues [81]. *FCGRT *haplotypes have been associated with the capacity to transfer IgG from cow to calf in beef cattle [82], which is of upmost importance in newborn, but not in adult animals on which the underlying phenotypic data of the discussed QTL studies are based on. Hence there is not sufficient evidence for the involvement of *FCGRT *polymorphism into the variation caused by the QTLs. An *IRF3 *polymorphism in mice alters induction of IFN-β response and affects resistance to *Listeria *infections [71]. Pathogens which use the same underlying mechanism of a pathogen-driven induction of IFN-β transcription to reduce the host's defence would probably also be affected by a similar polymorphism in *IRF3*.

All pathogens related to the QTLs in all analysed species can potentially be recognized by TLR3 or TLR4 which can activate an immune response via a MyD88 independent pathway resulting in activation of IRF3 [83]. In addition multiple other TLR independent pathways which are activated by pathogen recognition can result in activation of IRF3 [84]. Polymorphisms altering IRF3 transcript levels could therefore affect the resistance to a range of pathogens. This indeed was observed in resistant C57BL/6 mice compared to susceptible A/J mice nine days *post *infection with *Trypanosoma congolense *(Table 4).

For the other potentially relevant genes located in the homologous QTL regions (Table 4) to our knowledge no results suggesting an involvement of these genes in the QTL effects have been reported. Assuming, that one common gene is underlying the same QTL in sheep, cattle, mouse and human, *IRF3 *would be a compelling candidate.

#### *MyD88 *and linked genes

The QTL effect on the susceptibility to *Trypanosoma congolense *infections in cattle [43] and *Plasmodium chabaudi *infections in mice [54] might be related, as both diseases are the result of protozoan infections which presumably carry similar PAMPs and activate the same pathways. The chromosomal overlap of these QTLs suggests that they could be caused by the same genes in both species, while a connection with an overlapping QTL for *Borrelia burgdorferi *in mice is less obvious [51]. To date no evidence of differential expression has been reported for any of the six potentially QTL related genes (Table 4) in response to infections in pig, cattle or sheep. However, expression studies in mice show that three genes are differentially expressed in divergent mouse phenotypes *post *infection with *Trypanosoma congolense *[85] which includes *MyD88*, chemokine (C-X3-C motif) receptor 1 (*CX3CR1*), and chemokine binding protein 2 (*CCBP2*) and one *post *infection with *Yersinia enterocolitica *(*CX3CR1*) [86].

There are multiple chemokine receptors and ligands which are involved in the trafficking of leukocytes [87]. Although several of them are coded in the region around *MyD88 *in the so-called chemokine receptor cluster, the comparative genomic approach (Figure 2 and Table 4) excluded several of them due to their localisation. Polymorphisms in a number of chemokine receptors are associated with susceptibility and resistance to human immunodeficiency virus (HIV) infection [87]. The chemokine receptor genes have also been investigated as possible candidates for health traits in livestock [88]. However, to date no significant associations of the chemokine receptors located in the homologous QTL with protozoan infections have been detected.

In contrast, MyD88 is due to its central position as an adaptor molecule involved in the immune responses to many different pathogens, including protozoa (reviewed by Ropert *et al. *[72]). MyD88 has been associated with a protective effect during infection with *Trypanosoma *[89] and *Toxoplasma *[90] strains. Interestingly, during malaria infections MyD88 signalling is involved in an excessive cytokine production which is responsible for most of the clinical symptoms [72]. Thus, a hypothetical *MyD88 *polymorphism affecting the gene function could balance protection against different protozoan parasites. However, to date no evidence for such polymorphism is available. It can therefore concluded, that further investigations are required to elucidate the role of *MyD88*, *CX3CR1 *or *CCBP2 *in the variation caused by the QTL.

#### *TLR1 *gene family cluster and linked genes

The association of this chromosomal location with the susceptibility to bacterial infections in cattle (clinical mastitis) [38] and mice (*Listeria moncytogenes*) [48] is consistent with the function of TLR1 and 6 and polymorphisms within these genes have been associated with tuberculosis [70] and malaria [66] in humans. The association with malaria suggests together with the differential expression in divergent mouse, sheep and cattle phenotypes *post *infection with protozoan or other parasites (Table 4), that the *TLR1 *family cluster might also be involved in the recognition of further yet unknown ligands. The ligand for TLR10 is still unknown. However, TLR10 is not functional in mice and must therefore be excluded as a common candidate for both species, although it remains a possible candidate gene for the mastitis related QTL in cattle.

Another relevant gene, *RFC1 *(replication factor 1), had higher transcript levels in bone marrow-derived macrophages (BMDM) isolated from disease susceptible BALB/c mice than from resistant C57BL/6 mice *post *infection with *Yersinia enterocolitica*. This indicates that different variants might play a divergent role in the disease response. The *RFC1 *GO annotation points among others to its involvement in the defence [GO:0006952], which includes recovery functions such as DNA repair. However, to our knowledge no gene functions linking *RFC1 *directly with a mechanism which could be responsible for the trait variations are known and its low expression in resistant mice might simply reflect reduced requirement for DNA repair in more resistant animals. It can therefore be concluded that the *TLR1 *family gene cluster is the most likely candidate for the overlaying QTLs.

## Conclusion

A comparative approach enabled us to identify TLR-related genes in regions of conserved synteny among mammals that affect related traits in several species. We investigated their functional relevance for the trait in question, reviewed expression studies and analysed further immune related genes located in the regions. With the increasing availability of QTL and expression data, this approach could be extended to identify additional genes of economic interest in livestock and also to provide new insights into complex phenotypes in humans.

The genes involved in TLR signalling are suggested to be candidates for health traits in mammalian species. The most compelling evidence for involvement in pathogen susceptibility traits has been demonstrated for *TLR1, TLR6, MyD88 *and *IRF3*. Due to their close linkage and their functions or expression patterns some evidence suggests in addition *FCGRT*, *CX3CR1*, *CCBP2 *and *TLR10 *as further potential candidate genes. For *FCGRT*, *TLR1*, *TLR6 *and *TLR10 *SNPs have been established in pig [14] and cattle [82,91,92]. The other genes could be screened for SNPs which could then be tested for associations with health related traits in livestock.

The other TLR-related genes and further closely linked genes might be involved in mechanisms shaping immune related traits, although they were not considered here due to the limited availability of evidence. Additional investigations of polymorphisms in these genes should be pursued.

## Methods

### *In-silico *mapping using the pig FPC map

Positions of TLR-related genes in the pig genome were predicted using information of the porcine FPC map [27]. This integrated physical BAC map contains contigs constructed by fingerprinting and BAC end sequencing and is ordered using landmark maps and alignments with the human genome. The *in-silico *position for each locus was predicted by an alignment of the human genome sequence surrounding the localisation of the TLR-related gene [30] with the BAC end sequences in the FPC map. The reliability of this *in-silico *method was tested by RH mapping (see below) and BLAST analysis [93] against the emerging pig genome sequence [31].

### *In-silico *mapping using the virtual sheep genome

Positions of the TLR-related genes were predicted in sheep by identifying the gene sequences within the virtual sheep genome [32] using the virtual sheep genome browser [33]. These *in-silico *positions were tested by RH mapping (see below).

### Primer design for pig and sheep genes

Primers for the porcine signalling molecules and *TLR*s were designed from published sequences, including genomic and cDNA sequences (Additional file 3: Primers used for RH mapping of TLR and signalling molecules). Intron-exon boundaries were determined by aligning porcine cDNA sequences against either the partial pig genome sequence assembly (build SScrofa5) or against the bovine whole genome sequence assembly (build Btau 4.0), assuming conserved gene structures between both species.

Primer sequences derived from Connor *et al. *[23] for all TLR signalling genes but *MyD88 *were used for RH mapping in sheep. Oligonucleotides for the ovine *MyD88 *and the TLR genes were designed from ovine cDNA sequences. In order to identify intron-exon boundaries to facilitate primer design, bovine or ovine cDNA sequences were aligned with the bovine whole genome sequence assembly (build Btau 4.0).

All new primers were designed using Primer3 [94] with a targeted amplicon length of 300 bp. Other primers used for the development of RH maps for porcine chromosomes 8 and 13 were derived from the MARC v2 [28] and the PiGMaP consortium linkage map [95].

### RH maps for pig and sheep

#### Porcine radiation hybrid panel

DNAs from 94 cell lines of the 3000 rad porcine Cambridge-Roslin Radiation Hybrid panel [26] were amplified in order to establish presence or absence of the gene in each cell line. PCR was performed with the same touchdown program for all markers: 13 cycles with an initial annealing temperatures of 67°C, dropping by 0.5°C each cycle, followed by 24 further cycles with an annealing temperature of 60°C. Genomic ovine and hamster DNA were used as positive and negative controls, respectively. The amplification of each cell line was assessed by electrophoresis in 2.8% agarose gels. All reactions were conducted twice and scored independently by eye and/or by using GelScore software [96].

Resulting vectors (Additional file 5: RH vectors of markers used for mapping in pig and sheep) were assigned to chromosomes and two- and multi-point analysis were performed using Carthagene software [97]. Fifty-nine and 39 markers were included in the RH maps for SSC8 and SSC13, containing five and 14 linkage groups (LOD4), respectively. The marker order within each group was determined using the Default algorithm of Carthagene [97]. Groups were then ordered and orientated along the chromosomes using the order of common markers with the porcine MARC v2 map [28].

#### Ovine radiation hybrid panel

The 88 cell lines of the USUoRH 5000rad ovine radiation hybrid panel [34] were amplified as described above. The ovine RH maps were constructed using the rh_tsp_map 3.0 software package [98] and CONCORDE [99] linked with the QSopt package [100] as described [101,102]. Two-point RH linkage groups were constructed with a LOD of at least 5.0.

### Positions of genes in human, mouse and cattle

Positions of the analysed genes in the human (NCBI 36), mouse (NCBI m37) and cattle (Btau 4.0) genomes were retrieved from the ENSEMBL website [103] by name string-search.

### Definition of QTL overlaps

Markers limiting the significant QTL boundaries were identified in the relevant studies (Table 3) and their positions identified as described before. The genes limiting the QTL regions were then used to identify the homologous region in species with related QTLs. Genes located within the resulting homologous QTL overlaps were retrieved from the ENSEMBL database [103].

### Selection of candidate genes based on gene ontology annotation

Genes located within the homologous QTL overlaps and listed within the InnateDB non-redundant gene list [73] were considered as functionally relevant if their GO annotations contained the keywords "immune response", "cellular defence", "response to...(any pathogen)" or "defence to ... (any pathogen)".

### Analysis of expression data

Gene transcript data were retrieved from the corresponding databases and analysed for differential expression by calculating the ratio of transcript levels between populations. Differences between Means were tested by a two-tailed t-test using the corresponding Excel function. Only significantly different transcript levels (p < 0.05) were considered further.

## Authors' contributions

OJ designed the primers, screened the ovine and porcine RH panels, calculated the pig RH maps, built *in-silico *maps, performed the comparative QTL overlap study and prepared the draft manuscript. AK screened the porcine RH panel with microsatellite markers on SSC8. NLC screened the porcine RH panel with microsatellite markers on SSC13. SIA searched literature and murine microarray databases for evidence of TLR-related transcriptional response variation. KJ searched literature and bovine and ovine microarray databases for evidence of TLR-related transcriptional response variation. TAA searched literature and porcine microarray databases for evidence of TLR-related transcriptional response variation. HF built porcine *in-silico *maps. CW mapped RH vectors to the sheep RH map. NEC supervised ovine RH mapping and helped draft the manuscript. ALA supervised the pig RH project, conducted searches of the pig genome sequence and reviewed the manuscript, EJG designed and supervised the study and helped with drafting of the manuscript. All authors read and approved the final manuscript.

## Supplementary Material

Additional file 1 **Pig chromosome 8**. The file contains an RH map of porcine chromosome 8a and 8b (left) compared to the MARC v2 linkage map (Rohrer et al. [28], right). Common markers are connected by red lines. RH linkage groups (LOD4) are indicated by blue lines and the outer most markers of each group are indicated. TLR-related genes are boxed. Distances on the RH maps are indicated in cR and on the linkage map in cM. Click here for file

Additional file 2 **Pig chromosome 13**. The file contains an RH map of porcine chromosome 13 (left) compared to the MARC v2 linkage map (Rohrer et al. [28], right). Markers common to both maps are connected by red lines. RH linkage groups (LOD4) are indicated by blue lines and the extreme markers of each group are indicated. TLR-related genes are boxed. Distances on the RH map are indicated in cR and on the linkage map in cM. Click here for file

Additional file 3 **Primers used for RH mapping of TLR and signalling molecules**. The file contains the primer sequences for the mapped loci in sheep and pig. Click here for file

Additional file 4 **Immunologically relevant genes in regions of conserved synteny surrounding the *TLR1 *family cluster, *MyD88 *and *IRF3***. The file contains a list of genes located in the regions of conserved synteny which overlap with the discussed QTLs and which are listed in the innatedb gene list [73]. Ensembl IDs, gene names, murine orthologs, gene ontologies (GO term) and chromosomal localisation in human are given. Genes unique to mouse or murine genes for which the human orthologs are not listed in the innatedb gene list are itemized with their position in mouse. Genes considered as functionally relevant are highlighted by green background. Click here for file

Additional file 5 **RH vectors of markers used for mapping in pig and sheep**. The file contains RH vectors for each locus mapped in sheep and pig. Each position in the vector represents a cell line with "0" indicating no retention, "1" indicating retention and "2" indicating ambiguous results. Click here for file
