# Supplementary figures and images for: Comparative genomics of Toll-like receptor signalling in five species
Source: BMC Genomics. 2009 May 11;10:216. doi: 10.1186/1471-2164-10-216 (PMC2689273; doi:10.1186/1471-2164-10-216)

## Slide 1
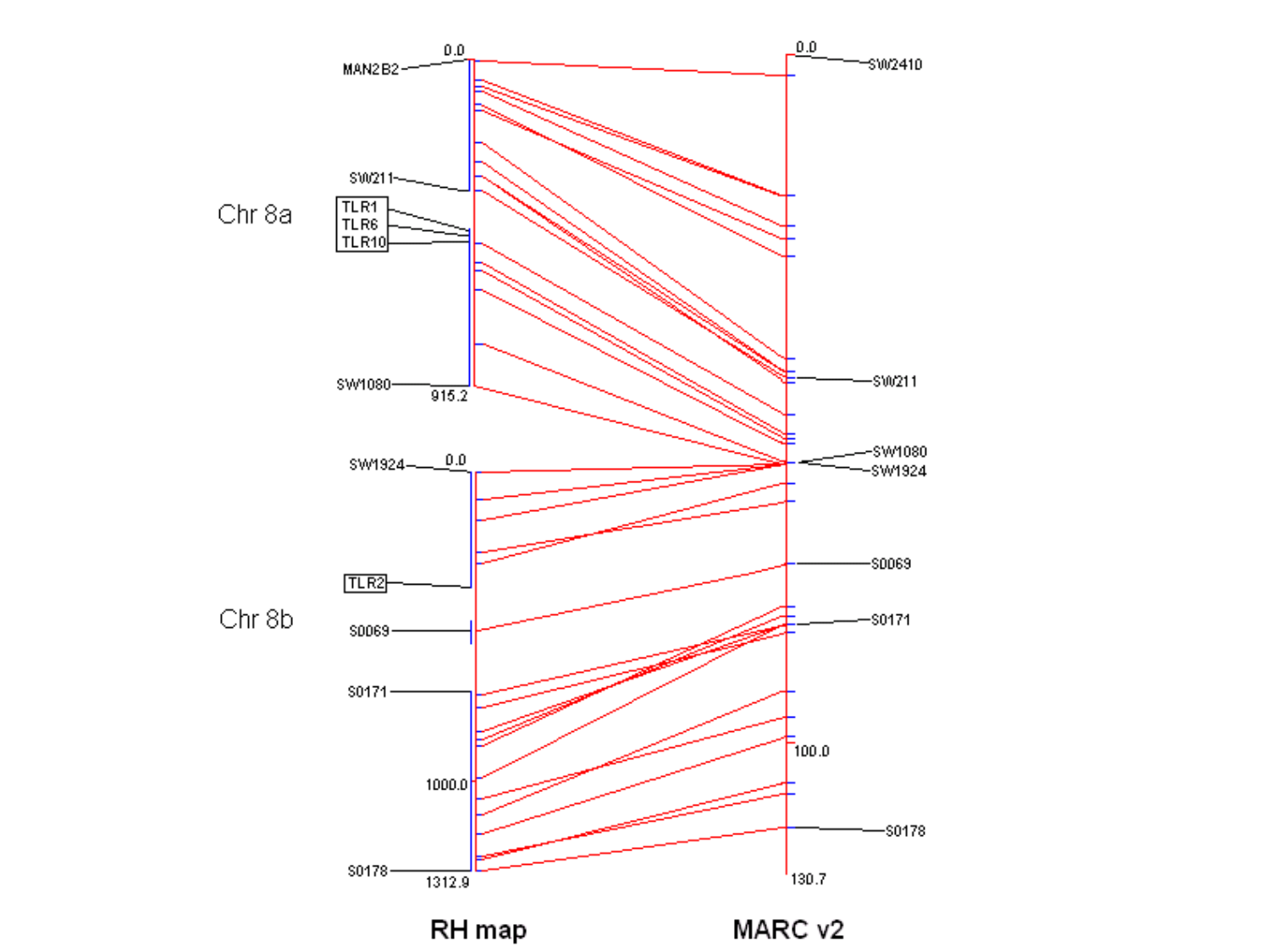

Supplement: Additional file 1 — Pig chromosome 8. The file contains an RH map of porcine chromosome 8a and 8b (left) compared to the MARC v2 linkage map (Rohrer et al. [28], right). Common markers are connected by red lines. RH linkage groups (LOD4) are indicated by blue lines and the outer most markers of each group are indicated. TLR-related genes are boxed. Distances on the RH maps are indicated in cR and on the linkage map in cM. [file 1471-2164-10-216-S1.ppt]

## Slide 1
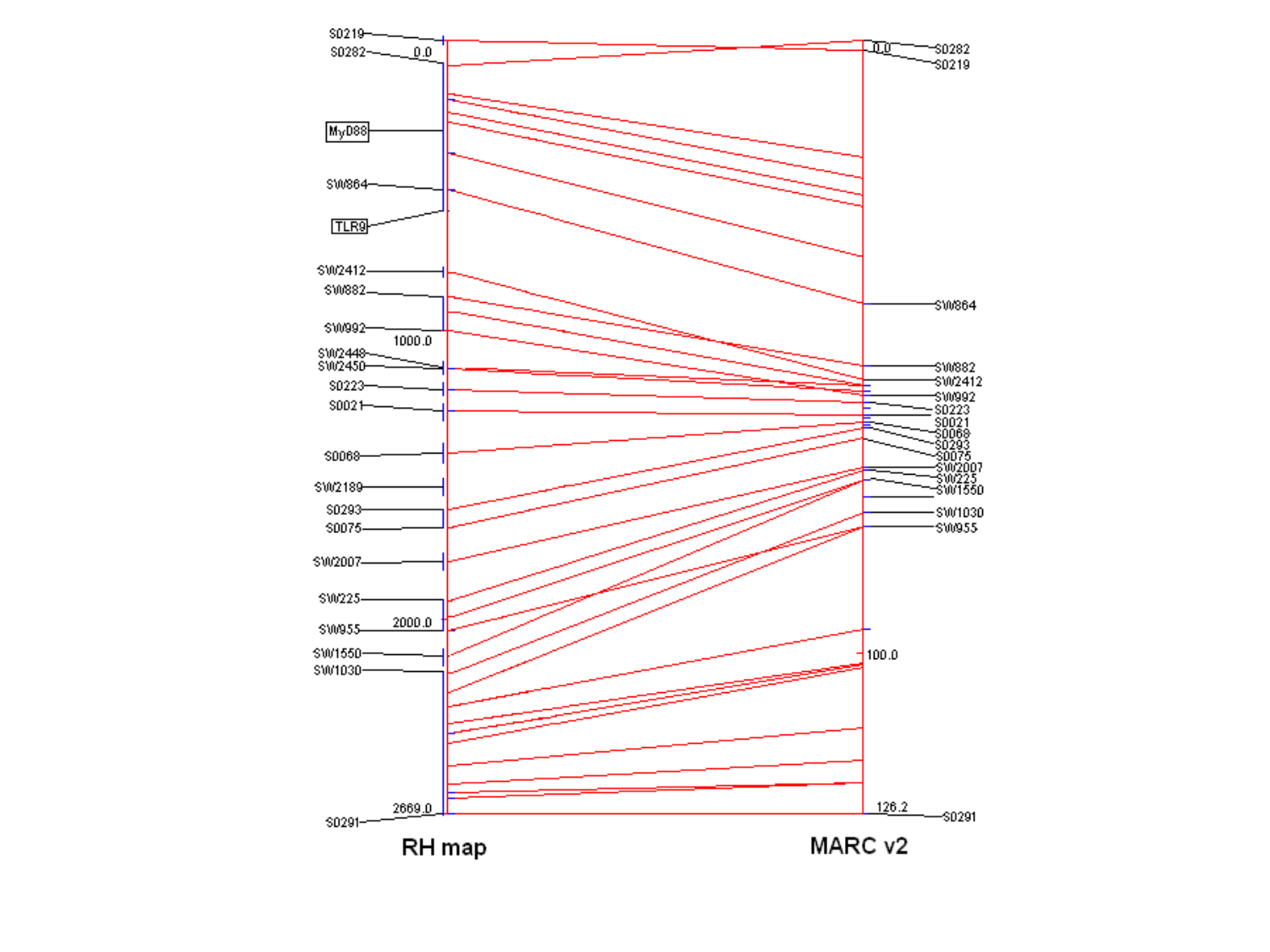

Supplement: Additional file 2 — Pig chromosome 13. The file contains an RH map of porcine chromosome 13 (left) compared to the MARC v2 linkage map (Rohrer et al. [28], right). Markers common to both maps are connected by red lines. RH linkage groups (LOD4) are indicated by blue lines and the extreme markers of each group are indicated. TLR-related genes are boxed. Distances on the RH map are indicated in cR and on the linkage map in cM. [file 1471-2164-10-216-S2.ppt]
